# Gut microbiome signatures during acute infection are associated with long COVID

**DOI:** 10.1080/19490976.2026.2718581

**Published:** 2026-08-19

**Authors:** Isin Y. Comba, Ruben A. T. Mars, Lu Yang, Mitchell Dumais, Jun Chen, Trena M. Van Gorp, Jonathan J. Harrington, Jason P. Sinnwell, Stephen Johnson, LaRinda A. Holland, Adam K. Khan, Efrem S. Lim, Christopher Aakre, Arjun P. Athreya, Georg K. Gerber, Jack C. O’Horo, Konstantinos N. Lazaridis, Purna C. Kashyap

**Affiliations:** a Division of Public Health, Infectious Diseases, and Occupational Medicine, Mayo Clinic, Rochester, MN, US; b Division of Gastroenterology and Hepatology, Mayo Clinic, Rochester, MN, USA; c Center for Individualized Medicine, Mayo Clinic, Rochester, MN, USA; d Department of Quantitative Health Sciences, Mayo Clinic, Rochester, MN, USA; e Department of Medicine, Mayo Clinic, Rochester, MN, USA; f Center for Fundamental and Applied Microbiomics, the Biodesign Institute, Tempe, Arizona, USA; g Department of Molecular Pharmacology and Experimental Therapeutics, Mayo Clinic, Rochester, Minnesota, USA; h Division of Computational Pathology, Brigham and Women's Hospital, Boston, MA, USA; i Health Sciences and Technology, Harvard University and MIT, Cambridge, MA, USA; j Harvard Medical School, Boston, MA, USA; k Division of Pulmonary and Critical Care Medicine, Mayo Clinic, Rochester, MN, USA; l Department of Physiology and Biomedical Engineering, Mayo Clinic, Rochester, MN, USA

**Keywords:** Long COVID, post-acute COVID-19 syndrome, microbiome, stool metagenomics, machine learning

## Abstract

**Background:**

Long COVID (LC) manifests in 10%–30% of non-hospitalized individuals post-SARS-CoV-2 infection, leading to significant morbidity. The predictive role of gut microbiome composition during acute infection in the development of LC is not well understood, partly because of the heterogeneous nature of the disease.

**Objectives:**

To determine whether the gut microbiome composition in the acute phase of SARS-CoV-2 infection predicts subsequent LC and to investigate the role of microbiome signatures in disease subphenotypes.

**Design:**

We conducted a longitudinal cohort study involving 799 outpatient participants tested for SARS-CoV-2 due to similar symptom presentation, including 380 SARS-CoV-2 positive and 419 negative individuals. Stool samples were collected at two time points for metagenomic sequencing. Logistic regression with L1 regularization was employed to predict LC based on the microbiome and clinical metadata.

**Results:**

The individuals who developed LC harbored a distinct gut microbiome during acute infection compared to those who recovered fully and uninfected controls with similar symptomatology. However, the temporal changes in the gut microbiome between the acute (0–1 month) and post-acute (1–2 months) phases were similar across the three cohorts. Using machine learning, we showed that the gut microbiome carried a modest signal for subsequent LC, but model performance was insufficient for clinical prediction, likely reflecting the heterogeneous nature of LC. Finally, we identified four LC symptom clusters, with gastrointestinal and fatigue-only groups strongly linked to gut microbiome alterations.

**Conclusion:**

The gut microbiome can potentially offer solutions for understanding the heterogeneous nature of LC. Larger cohorts and phenotype-aware computational algorithms may help overcome current model performance limitations and support the development of targeted diagnostic and therapeutic strategies.

## Introduction

Long COVID (LC) affects approximately 10%–30% of non-hospitalized individuals infected with SARS-CoV-2, with nearly 18% of U.S. adults reporting a previous history.^1^ ^,^ ^2^ This condition inflicts significant morbidity, workforce loss, and an estimated economic impact of $3.7 trillion in the U.S.^3^ LC manifests through a broad spectrum of persistent symptoms and host physiological changes, ranging from cardiovascular and gastrointestinal disturbances to cognitive impairments and neurological signs and symptoms.

Notably, these symptoms are similar to those of myalgic encephalomyelitis/chronic fatigue syndrome and other post-infectious syndromes, underscoring their common clinical presentation.^2^ ^,^ ^4^ ^,^ ^5^ The pathobiology of LC is complex and not well understood, complicated by the disease's heterogeneity and emerging evidence of distinct clinical subphenotypes.^6-8^ Proposed underlying mechanisms for LC include reduced peripheral serotonin levels,^9^ exaggerated humoral immune responses,^10^ coagulation abnormalities,^11^ neuroinflammation, persistence of viral antigens,^2^ and the presence of SARS-CoV-2 in reservoir tissues.^12^ The gut microbiome's established role in shaping both innate and adaptive immunity,^13^ its influence on antiviral immunity via inflammasome pathways in respiratory infections such as influenza A,^14^ and may thus be of importance in LC pathogenesis.

Prior studies, primarily conducted in hospitalized patients, have identified associations between LC and gut microbiome composition during acute infection and up to 6 months post-infection.^15-20^ These investigations have provided valuable insights, especially into the effects of severe illness. However, microbiome alterations observed in hospitalized patients may be influenced by physiological stress, medications, and clinical interventions-factors that are difficult to disentangle due to the inherent challenges of identifying well-matched control groups with comparable exposures.^21^ ^,^ ^22^ As the profile of long COVID shifts toward predominantly outpatient populations with milder illness, studying these individuals has become increasingly important, as they likely represent a broader and more representative spectrum of those now at risk for LC.

To address this gap, we designed a study that includes outpatients diagnosed with COVID as well as contemporaneous controls presenting with new-onset symptoms who tested negative for COVID. In this study, we show that the gut microbiome during acute infection is associated with the development of LC, and distinct microbes are associated with symptom-based LC clusters. The ability to identify patients who are likely to get LC is crucial for advancing our understanding of its pathobiology, the development of targeted diagnostic tools, and microbiome-based therapies to alleviate chronic symptoms.

## Materials and methods

### Research ethics approval

The study involves human participants and was reviewed and approved by the Mayo Clinic Institutional Review Board #20-005988.

### Study design and population

We recruited adults aged 18 y or older who underwent SARS-CoV-2 respiratory testing at Mayo Clinic’s multi-site health system in Minnesota, Florida, and Arizona, from October 2020 to September 2021. Potential participants were identified by reviewing daily reports from electronic health records (EHRs) filtered by SARS-CoV-2 testing (both PCR and NAAT) and scheduling orders. Only provider-ordered tests scheduled and performed in an outpatient setting were considered for inclusion, excluding individuals with self-reports and home test kit results. After confirming eligibility of age (18+), capacity to consent (per filing of legal authorized representatives or power of attorney), females not currently pregnant (at the time of medical record review), and confirmed scheduled SARS-CoV-2 test, subjects were contacted via a recruitment email. Informed consent was obtained from each participant. Patients and the public were not involved in the design, conduct, reporting, or dissemination plans of the above research.

### Specimen collection and data generation

#### Stool sample collection

Participants were asked to submit two stool samples at week 0–2 and week 3–5 after the initial SARS-CoV-2 test. Home collection kits, along with detailed collection instructions, were mailed to each participant. Upon collection, samples were returned in tubes on frozen gel packs via overnight FedEx service. Stool samples returned within 31 d of the index date were classified as Timepoint 1 (T1) samples, and those received afterward as Timepoint 2 (T2) samples. T1 represents acute illness (0–1 month) and was used for the majority of downstream analyses. Stool samples were stored at −80°C upon receipt, transferred to −20°C overnight before aliquoting, and then aliquoted at Mayo Clinic under sterile conditions into study-specific aliquots for downstream analyses, including DNA extraction/sequencing, fecal calprotectin, and SARS-CoV-2 E gene qRT-PCR.

#### Microbial DNA extraction, sequencing, and taxonomic assignment

Stool aliquots were sent to the University of Minnesota Genomics Center. DNA extraction from each sample was performed using Qiagen's DNeasy 96 PowerSoil Pro QIAcube HT Kit following the manufacturer's instructions. Metagenomic libraries were prepared using the Nextera XT protocol. Samples were sequenced on a NovaSeq 6000 (Illumina, San Diego, CA, USA), targeting 8 million reads per sample (2 × 150 bp). Taxonomic profiling of reads was performed using Kraken2 with the GTDB v202 database.^23^ Relative abundances were then estimated using Bracken.^24^ Functional profiling of metagenomic data was performed using HUMAnN3.^25^

#### Stool SARS-CoV-2 RNA detection

200  mg of stool was added to 1.7 ml of PBS and vortexed for 3 min at 3,000 × g. The samples were centrifuged at 20,000 × g for 5 and then 1  ml of supernatant was transferred into a bioMérieux eMAG (Marcy-l’Étoile, France) for total nucleic acid extraction. Detection of SARS-CoV-2 was performed by TaqMan-based RT-qPCR using SuperScript III Platinum One-Step RT-qPCR Kit (Invitrogen, Carlsbad, CA) and E-Sarbeco primers and probe as previously described.^26^ A 25 µl total reaction of 4.5 µl molecular grade water, 12.5 µl of SSIII 2x-MasterMix, 1 µl of each 10 µm primer, 0.5 µl of 10 µm probe, 0.5 µl of SSIII Taq, and 5 µl of TNA. Cycling conditions on a Quant Studio 7 were a 50° RT step for 5 min, a single denaturation of 95° for 2 min, followed by 45 cycles of 95° for 3 s and 58° for 30 s.

#### SARS-CoV-2 genome sequencing

SARS-CoV-2 genome sequencing was performed on samples that were SARS-CoV-2 RNA-positive using the COVIDSeq Test (Illumina, San Diego, CA, USA). Libraries were sequenced on the Illumina NextSeq2000 instrument using 2 × 10^9^ paired-end reads. Sequencing reads adapter sequences were trimmed using trim-galore, aligned to the Wuhan1 reference genome (MN908947.3) using the Burrows–Wheeler aligner, BWA-MEM version 0.7.17-r1188, and primer sequences trimmed using iVAR version 1.3.1. SARS-CoV-2 lineage calling was performed using Pangolin.^27^

#### Stool calprotectin measurement

Calprotectin in stool was measured at Arizona State University using the BÜHLMANN fCAL ELISA kit (BÜHLMANN Diagnostics Corp, Amherst, NH) per the manufacturer's instructions. For the extraction preparation, ~30mg of stool was weighed and transferred into a calprotectin assay tube. The samples were diluted 1:50 with the kit extraction buffer based on input weight, then vortexed for 3 min at 3,000 × g. The samples were then heat-inactivated at 65°C for 30 min. Following heat inactivation, the samples were centrifuged at 3,000 × g for 5 min. The supernatant was transferred to Eppendorf tubes and stored at −80°C until further analysis. A four-parameter logistic function, calibrated with five standard samples in each assay, was used to convert sample optical density to calprotectin concentration. Concentrations were normalized to adjust for varying stool dilution volumes. Based on the clinical threshold guidelines provided, the calprotectin status of each sample was categorized as normal (<80 μg/g), gray zone/borderline (80–160 μg/g), or elevated (>160 μg/g).

#### Clinical data acquisition and case definitions

LC was defined as the persistence of symptoms for 3 months or more after the onset of illness, or new-onset symptoms not explained by an alternate diagnosis.^28^ The index date was defined as the SARS-CoV-2 test date for both SARS-CoV-2-positive and -negative groups. To ascertain symptom status and types experienced, a prospective review of EHRs, including provider notes, SARS-CoV-2 testing orders, nurse screening forms, and patient portal communications, was conducted for at least 1 y from the index date. Two primary reviewers adjudicated LC status (M.D. and I.Y.C.). The third and fourth reviewers (J.C.O. and P.C.K.) were consulted to achieve consensus when needed. Demographics, comorbidities, medications, hospitalization, and laboratory results were digitally extracted from the Mayo Clinic Unified Data Platform and our EHR system (Epic Systems, Inc., Verona, WI) using rapidSQL (Idera, Inc., Austin, TX). Manual chart reviews were conducted to confirm SARS-CoV-2 test date and reason for testing, last clinic encounter date, index COVID-19 disease severity, complications, comorbidities (e.g., inflammatory bowel disease, immunosuppression, solid organ transplantation), and 30-day use history of antibiotics, proton pump inhibitors, or immunosuppressive medications. Further information on the antibiotic type, duration, route of administration, and reason for use was obtained. Data were stored and managed using REDCap electronic data capture tools hosted at Mayo Clinic.^29^

We classified COVID-19 vaccination status as fully vaccinated, first dose, and unvaccinated. Patients were considered fully vaccinated two weeks after they completed a two-dose mRNA COVID-19 live attenuated vaccine series (BNT162b2 [Pfizer-BioNTech], mRNA-1273 [Moderna]) or a single-dose viral vector live attenuated vaccine (Ad26.COV2.S [Janssen]).^30^ COVID-19 disease severity was adopted from the NIH COVID-19 treatment guidelines and consisted of five categories: (1) asymptomatic or pre-symptomatic, (2) mild illness, (3) moderate illness, (4) severe illness, and (5) critical illness.^31^ Comorbidities were classified and standardized using the unweighted Charlson Comorbidity Index (CCI) score using the “comorbidity” package in R (v4.3.3; R Core Team 2024).^32^ ^,^ ^33^

### Statistical analysis

#### Microbiome data analysis

Microbial alpha and beta diversity analyses were conducted using the R “vegan” (v2.6-6.1), “GUniFrac” (v1.8) and “phyloseq” (v1.46.0), packages after rarefaction.^34-36^ Chao1 species richness estimator and Shannon indices were used for alpha diversity analysis, while Bray-Curtis (BC) dissimilarities and generalized UniFrac (GUniFrac) distance were used for beta diversity analysis. One-way ANOVA and PERMANOVA were used for testing the group association with alpha and beta diversity, respectively.^35^ Analysis was adjusted for clinical variables that were statistically different (*p* < 0.05) between comparison groups to control for confounding effects. Statistical significance for diversity analysis was set at *p* < 0.05. For post hoc analysis of beta diversity, “RVAideMemoire” (v0.9-83-7)^37^ was employed for pairwise comparisons with false discovery rate (FDR) correction. To investigate the relationship between microbial diversity and LC group membership, redundancy analysis (RDA), a form of constrained ordination, was conducted using the “vegan” package.^34^ Redundancy analysis involved assessing a matrix of interval-scaled data (species relative abundances) against a matrix of covariates through singular value decomposition. Procrustes analysis was conducted on longitudinal microbiome data to inspect any changes in microbial beta diversity between T1 and T2 samples.^38^ To quantify and compare the magnitude of microbiome changes over time across three cohorts, we extracted pairwise Procrustes by sample and calculated Euclidean distances. Differential taxa abundance analysis was performed using a linear model-based permutation procedure in the “ZicoSeq” function of the “GUniFrac” package.^35^ ^,^ ^39^ The FDR-adjusted *p*-value threshold was set at 0.1 (i.e., allowing on average 10% false discoveries in the results to identify taxa with moderate effects).

### Software and algorithms

#### Applying machine learning models to predict LC

Logistic regression with L1 regularization was employed to model LC in our study population. The goal of the model was to classify LC (*n* = 80) and non-LC (*n* = 300) from the SARS-CoV-2-positive cohort, using one sample (T1) per individual. Input data for models included species-level or genus-level relative abundance matrices and/or clinical covariates. The clinical covariates used as input for the models included age, sex, BMI, CCI scores, index COVID-19 disease severity, SARS-CoV-2 vaccination status, proton pump inhibitor use, immunosuppressive medication use, 30 d antibiotic use, individual comorbidities such as chronic pulmonary disease, diabetes mellitus, chronic kidney disease, inflammatory bowel disease, myocardial infarction, metastatic cancer, moderate-to-severe liver disease, and rheumatologic disease, transplant history (solid organ or bone marrow transplantation) and detailed use of immunosuppressive medication use including glucocorticoids, active cancer chemotherapy, calcineurin inhibitors, antimetabolites, biologics, antiproliferative agents, and other immunosuppressive therapies. Class weights were calculated using “scikit-learn” (v1.2.2) based on the frequencies of labels (0, 1), with weights assigned inversely proportional to prevent bias toward overrepresented classes.^40^ Model development and validation were performed using nested cross-validation with an architecture of an inner loop for hyperparameter tuning using “GridSearchCV” function^40^ and an outer loop for validating each model’s performance. This setup was repeated 10 times on random seeds for reproducibility. Five-fold cross-validation was applied to the inner and outer loop and performance metrics, including area under the receiver operating characteristic (AUROC), weighted F1 score, precision, and recall, which were calculated for each model configuration to select the optimal parameters. All models were developed using Python (Python Software Foundation, Beaverton, OR).

#### Modeling LC disease heterogeneity

To identify potential disease subphenotypes in LC, 18 symptoms observed in more than 10% of LC patients were extracted. Multiple correspondence analysis (MCA) was conducted to reduce its dimensionality and derive coordinates using “factoextra” (v1.0.7),^41^ and the resulting MCA factor plot (Supplemental Figure 1a) was examined to assess data structure and discernible clustering tendencies. A heatmap^42^ with hierarchical and *k*-means clustering (*n* = 999) was then generated on the symptom matrix, using a binary dissimilarity metric to further delineate symptom clusters.

To determine the optimal number of clusters (*k*), a binary distance matrix was computed, followed by classical multidimensional scaling (MDS) to reduce the noise in the data observed in MCA plots. The first three MDS dimensions, accounting for 47% of total variance, were used to calculate the within-cluster sum of squares (WCSS) for *k* from 2 to 10. An elbow was observed at *k* = 4 (Supplemental Figure 1b), balancing cluster coherence and parsimony. To further support the findings with elbow technique, mean silhouette widths s(*i*), were calculated for iterated *k* values (2–10) using “cluster” (v2.1.6)^43^ package, which showed a peak at *k* = 4 (Supplemental Figure 1c).

### Reporting statement

This study was reported in accordance with the STROBE (Strengthening the Reporting of Observational Studies in Epidemiology) guidelines for cohort studies.^44^ Extended methods and materials are available in supplementary materials.

## Results

### Study population baseline characteristics

Our study analysis comprised 947 stool samples from 799 subjects – 380 testing positive and 419 testing negative for SARS-CoV-2. Within the follow-up period, 80 patients who were positive for SARS-CoV-2 developed LC; hence, for downstream analysis, we categorized SARS-CoV-2-positive patients as LC (SARS-CoV-2-positive patients who subsequently developed LC) or non-LC (SARS-CoV-2-positive patients who did not develop LC). Electronic medical records were similarly reviewed in the SARS-CoV-2-negative group to identify recurrent or persistent symptoms not attributable to pre-existing comorbidities. 16 of 419 participants lacked adequate clinical follow-up to assess symptom resolution. Among the remaining 403 participants, 12 (3%) had recurrent or persistent symptoms. These symptoms were heterogeneous, with isolated atypical chest pain being the most frequent persistent presentation (*n* = 3).

**Figure 1.**
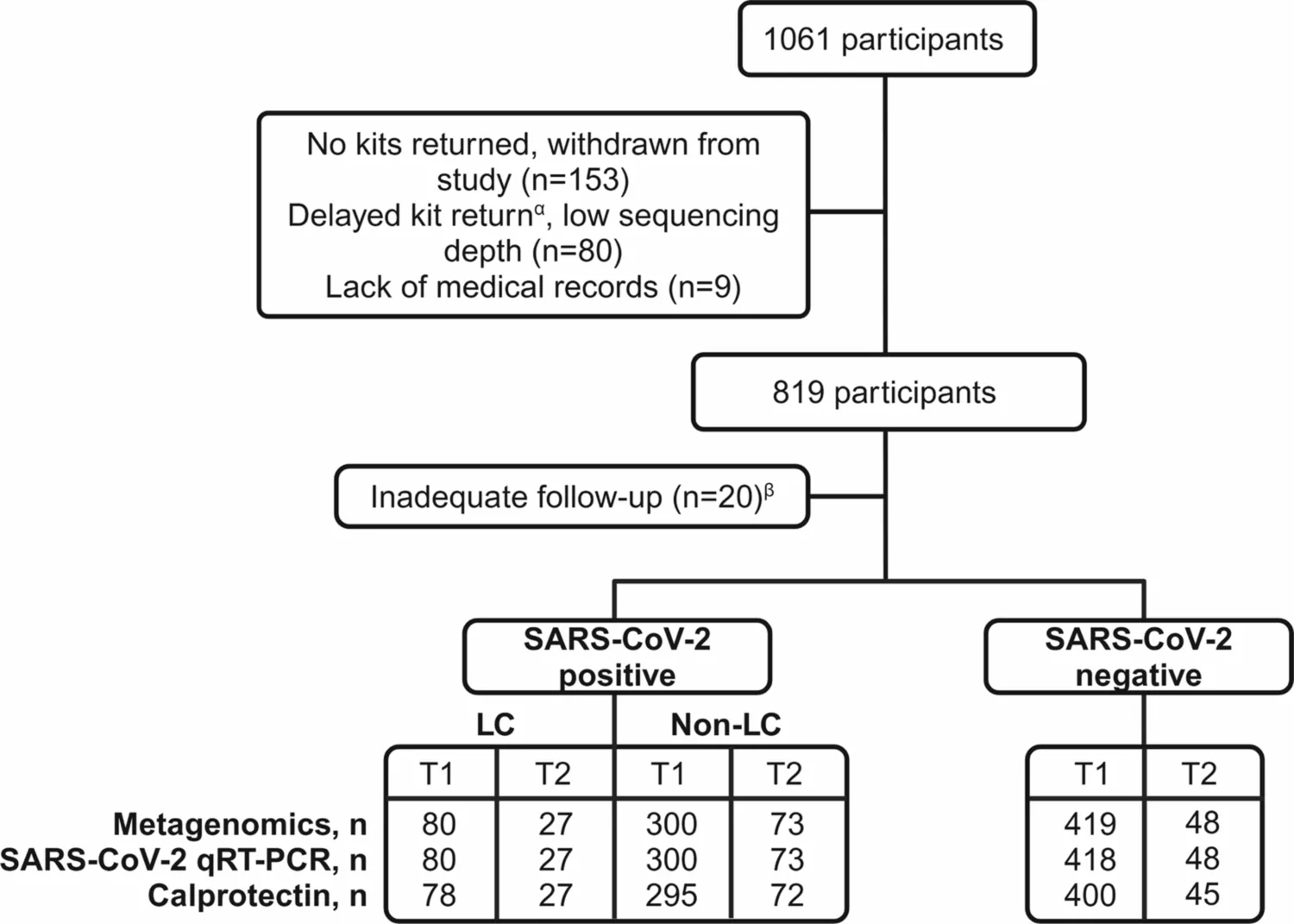
Overview of study design and number of fecal samples analyzed. Our study included three groups: Long COVID (LC), SARS-CoV-2-positive but non-long COVID (non-LC), and SARS-CoV-2-negative. Fecal samples were collected at two timepoints after the initial SARS-CoV-2 test date, denoted as T1 and T2, and were analyzed as indicated. *α*, The first kit was received 31 d after the initial SARS-CoV-2 test date or only second kit is returned. *β*, Patients in the SARS-CoV-2 positive arm with inadequate follow-up to ascertain LC status were excluded from further analysis.

The study enrollment process and cohort census are detailed in the methods and Figure 1. Baseline characteristics of SARS-CoV-2-positive (both LC and non-LC) and -negative participants are outlined in Table 1 and Supplemental Table 1. Post hoc analysis showed a higher proportion of females in the LC group than in other groups (pairwise Fisher's exact test, *p* < 0.05) and a higher number of baseline comorbidities (Dunn’s test, Bonferroni correction, *p* = 0.009) compared with non-LC. The SARS-CoV-2-negative group was significantly older (Tukey's HSD, *p* < 0.001) and had a higher rate of 30-day antibiotic use (17% vs. 6%, pairwise Fisher's exact test, *p* < 0.001) compared with non-LC and higher vaccination rates (pairwise Fisher's exact test, *p* < 0.001) than other groups. No significant differences in body mass index (BMI) or race/ethnicity were observed across groups. In the SARS-CoV-2-negative group, 56% of patients were symptomatic, with sore throat (32%), cough (31%), and headache (31%) as the most common symptoms. On the other hand, 89% of the non-LC group and 99% of the LC group were symptomatic, presenting predominantly with cough (Supplemental Table 2). Among symptomatic SARS-CoV-2-negative participants, 90 underwent provider assessment and/or additional diagnostic evaluation that identified a possible alternative etiology. In contrast, 144 participants (61.5%) had no documented specific etiology. This group included 121 participants who did not receive provider assessment directed at their reported symptoms, 13 who had no longitudinal follow-up, and 10 who reported symptoms but underwent SARS-CoV-2 testing for screening purposes rather than because of those symptoms (Supplemental Table 3). The median time from index test to T1 stool kit return was 12 d [IQR, 10–15], and 89.4% of T1 samples were collected within 15 d of the test (Supplemental Figure 2).

**Table 1.** Baseline characteristics of the study population.

	LC (*n* = 80)	Non-LC (*n* = 300)	SARS-CoV-2-negative (*n* = 419)	*p*
Age, y, mean (SD)	56 (13)	56 (15)	60 (13)	<0.001
Female, *n* (%)	61 (76)	169 (56)	254 (61)	0.005
BMI, kg/m^2^, mean (SD)	29.1 (6.4)	28.6 (5.5)	28.2 (6.4)	0.4
Race/ethnicity, *n* (%)				
Hispanic (all races)	4 (5)	9 (3)	15 (4)	
White, non-Hispanic	69 (86)	283 (94)	376 (90)	
Black, non-Hispanic	3 (4)	2 (1)	10 (2)	
Asian, non-Hispanic	2 (2.5)	3 (1)	15 (4)	
All other, non-Hispanic	2 (2.5)	2 (1)	2 (0.5)	
Choose not to disclose/missing	–	1 (0.3)	1 (0.2)	
CCI score, median (IQR)	1 (0–2)	0 (0–1)	0 (0–2)	0.006
Symptomatic, *n* (%)	79 (99)	268 (89)	234 (56)	<0.001
Thirty-day antibiotic use, *n* (%)	9 (11)	17 (6)	70 (17)	<0.001
Fully vaccinated, *n* (%)	18 (23)	95 (32)	298 (71)	<0.001

Abbreviations: BMI, body mass index; CCI, Charlson Comorbidity Index; IQR, interquartile range; SD, standard deviation. CCI scores and time to kit receipt were analyzed using the Kruskal–Wallis test, with post hoc assessment via Dunn’s test with Bonferroni correction. Age and BMI were evaluated with an unbalanced one-way ANOVA, and age differences were further analyzed using Tukey’s HSD. Sex, 30-day antibiotic use, and vaccination status were assessed with Fisher’s exact test, followed by pairwise Fisher's exact test with Holm correction for post hoc analysis. In the SARS-CoV-2-negative group, BMI for five patients, antibiotic use, and CCI scores for three patients were missing.

### Long COVID patients harbor a distinct gut microbiome during acute infection

**Figure 2.**
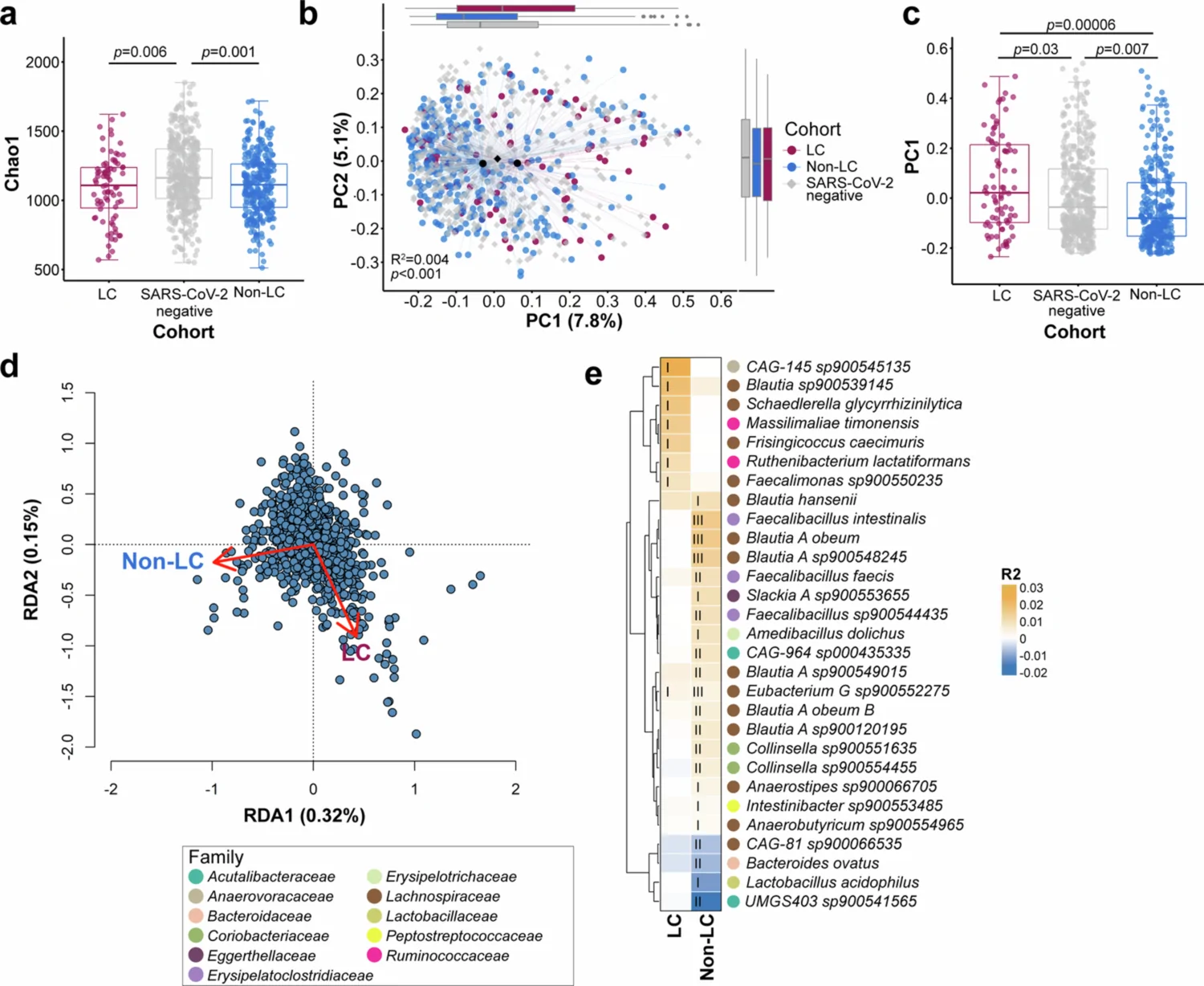
LC patients harbor a distinct gut microbiome compared with non-LC and SARS-CoV-2-negative individuals. (a) Alpha diversity plotted using the Chao1 index. (b) Principal coordinate analysis (PCoA) of species-level Bray–Curtis (BC) distances of LC (*n* = 80) vs. non-LC (*n* = 300) subjects and SARS-CoV-2-negative controls (*n* = 419) adjusted for age, sex, baseline Charlson Comorbidity Index (CCI) score, chronic pulmonary disease, and antibiotic use. (c) Box plots of the first principal coordinate (PC1) values derived from PCoA. (d) Redundancy analysis (RDA) reveals the directionality and effect size of LC and non-LC group memberships on species-level microbial diversity of samples collected during acute infection. (e) Heatmap displaying differential species-level abundances between SARS-CoV-2-negative controls (*n* = 419, reference) and LC patients (*n* = 80, adjusted for age and sex) or non-LC patients (*n* = 300, adjusted for age, CCI score, and antibiotic use). Levels of statistical significance are represented by Roman numerals: I, FDR-adjusted *p* < 0.1; II, *p* < 0.05; and III, for *p* < 0.01. In panels a and c, box plots indicate the median (central line) with whiskers extending to the 5th and 95th percentiles.

Alpha diversity based on the Chao1 metric is significantly lower in SARS-CoV-2-positive patients (both LC and non-LC) compared to SARS-CoV-2-negative subjects, but comparable based on the Shannon index (Figure 2a **,** Supplemental Figure 3a). PCoA based on Bray–Curtis (BC; PERMANOVA, R^2^ = 0.004, *p* < 0.001; Figure 2b) and GUniFrac (PERMANOVA, R^2^ = 0.004, *p* = 0.002; Supplemental Figure 3b) beta diversity metrics show significant differences in microbial composition among study cohorts (LC, non-LC, and SARS-CoV-2-negatives) after adjusting for clinical age, sex, baseline comorbidity score, chronic pulmonary disease and antibiotic use. Post hoc analysis reveals that patients who develop LC have a distinct gut microbiome profile during acute infection compared with those who do not develop LC and SARS-CoV-2-negative subjects (pairwise comparisons using PERMANOVA, FDR-adjusted *p* < 0.05). This distinction in microbial diversity was particularly pronounced along the PC1 axis between LC and non-LC cohorts (Tukey’s HSD, *p* = 0.00006; Figure 2c). Furthermore, during the acute infection phase, the LC and non-LC cohorts exhibited changes in microbial diversity along two distinct RDA axes, highlighting gradients of diversity shifting in opposite directions (Figure 2d). At the taxonomic level, compared to SARS-CoV-2-negative individuals, both LC and non-LC cohorts exhibited distinct gut microbiome profiles, characterized by minimal overlap in significantly altered taxa (ZicoSeq, FDR-adjusted *p* < 0.1; Figure 2e).

Abundances of microbial genes assorted into pathways significantly differed among the three groups (BC dissimilarities, PERMANOVA, R^2^ = 0.009, *p* = 0.002), but these differences diminished after adjusting for clinical confounders (R^2^ = 0.004, *p* = 0.085). Compared to SARS-CoV-2 negative controls, the non-LC cohort exhibited significant reductions in 13 microbial pathways and enrichments in 3 pathways (ZicoSeq, FDR-adjusted *p* < 0.1; Supplemental Figure 4a). Specifically, pathways related to nitrogen cycling and amino acid metabolism – including the urea cycle, anaerobic purine nucleobase degradation II, L-histidine degradation III, and L-citrulline biosynthesis – were significantly decreased in non-LC individuals. In contrast, pathways involved in carbohydrate degradation and cofactor biosynthesis demonstrated variable trends between non-LC and SARS-CoV-2 negative cohorts, while no statistically significant differences in mapped and integrated microbial pathways were observed between LC and SARS-CoV-2 negatives (Supplemental Figure 4a, b).

Fecal calprotectin, a well-established biomarker for gut inflammation and neutrophil activity, is a potential indicator of these signs in COVID-19 patients.^45^ ^,^ ^46^ However, in our cohort, fecal calprotectin levels were not associated with SARS-CoV-2 positivity or LC at any study phase (Fisher's exact test, *p ≥* 0.7 for both time points; Table 2). During acute infection, the PCR detection rate of the SARS-CoV-2 E gene in stool samples was 11% for the SARS-CoV-2-positive cohort (LC and non-LC), with 7.5% and 12% positivity rates in the LC and non-LC groups, respectively. We also saw no association between fecal PCR positivity and calprotectin level (Fisher's test, *p* = 0.55). Among the successfully sequenced SARS-CoV-2-positive samples, B.1.2 (*n* = 11) was the most prevalent lineage, followed by B.1.617.2 (delta variant, *n* = 5), and AY.103 (*n* = 3) (Supplemental Table 4).

**Table 2.** Qualitative stool calprotectin levels and SARS-CoV-2 qRT-PCR characteristics.

	Timepoint-1				Timepoint-2			
	LC (*n* = 80)	Non-LC (*n* = 300)	SARS-CoV-2 negative (*n* = 419)	*p*	LC (*n* = 27)	Non-LC (*n* = 73)	SARS-CoV-2 negative (*n* = 48)	*p*
Calprotectin, µg/gm				0.8				0.7
< LOD/Normal (<79.9)	53 (66)	198 (66)	280 (67)		23 (85)	60 (82)	40 (83)	
Borderline (80–160)	9 (11)	39 (13)	40 (9.5)		4 (15)	12 (16)	5 (10)	
Elevated/above calibration curve (>160.1)	16 (20)	58 (19)	80 (19)		0	0	0	
Missing	2 (2.5)	5 (2)	19 (4.5)		0	1 (1)	3 (6)	
SARS-CoV-2 E gene stool qRT-PCR								
Detected	6 (7.5)	35 (12)	3 (1)		0	1 (1)	0	
Undetected	74 (92.5)	265 (88)	415 (99)		27 (100)	72 (99)	48 (100)	
Missing	0	0	1 (0.2)		0	0	0	
SARS-CoV-2 E gene CT value, median (range)	32 (27–35)	32 (18–37)	35 (34–36)		–	–	–	

The level of significance was calculated using Fisher’s exact test. Abbreviations: CT, cycle threshold; LOD, level of detection; qRT-PCR, reverse transcription polymerase chain reaction.

### Gut microbiome composition does not change significantly over time at the cohort level, independent of LC status

**Figure 3.**
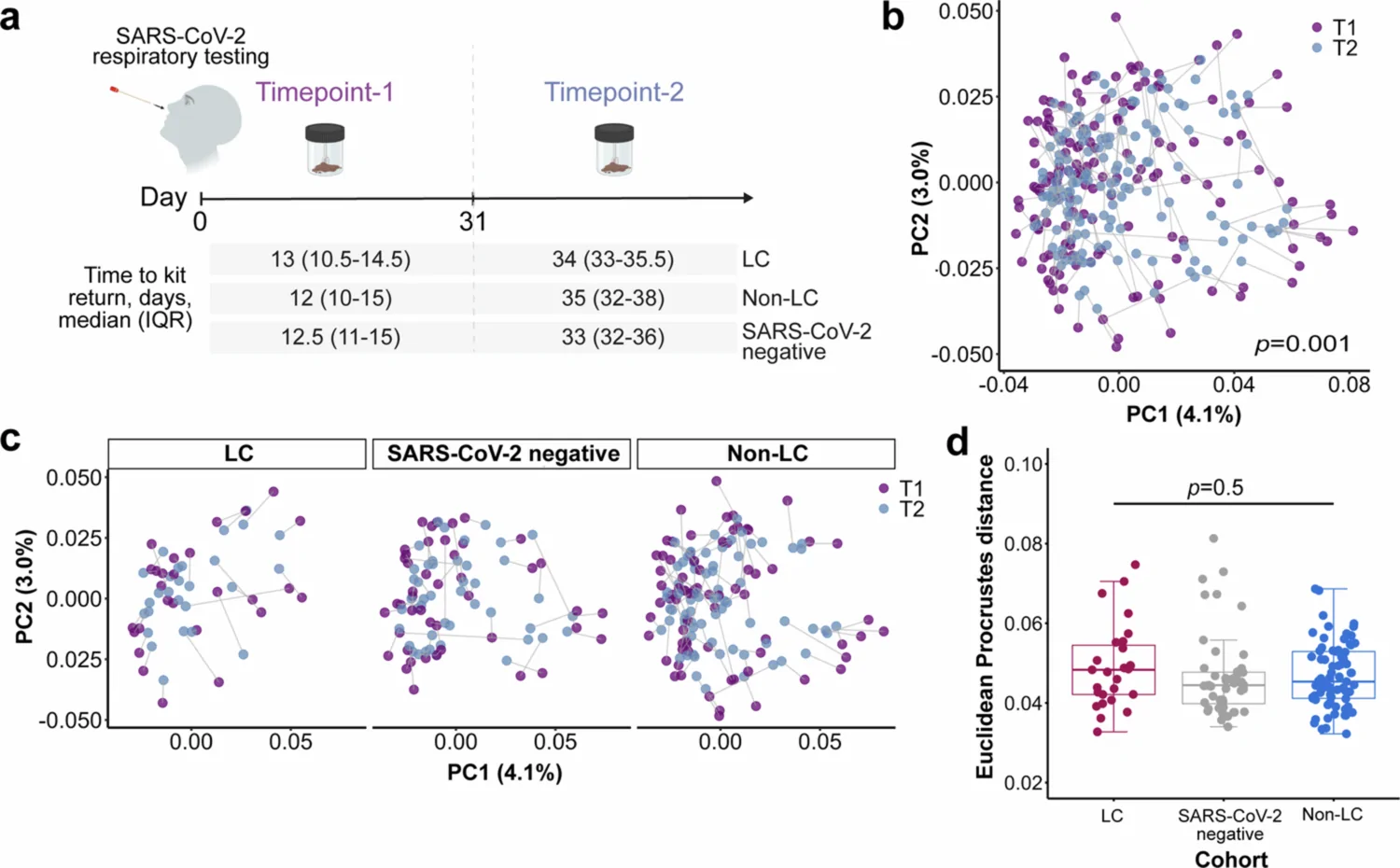
Longitudinal gut microbiome variability across study cohorts. (a) Overview of stool sampling timelines and median return times for kits. PCoA plots using Bray–Curtis dissimilarities were generated to compare microbial communities within individual subjects (b) and across different cohorts (c) between the two sampling timepoints. Timepoints 1 and 2 samples from the same subject are interconnected by lines. Procrustes analysis showed statistically significant similarity in the spatial arrangement of the microbial communities between timepoints 1 and 2 samples (*p* = 0.001). (d) Boxplots illustrating Euclidean distances calculated from pairwise Procrustes distances across three study cohorts. No statistically significant differences were noted (Tukey’s HSD *p* ≥ 0.5 for all comparisons). Number of patients: LC (*n* = 27), non-LC (*n* = 73), and SARS-CoV-2-negative (*n* = 48). Median values and interquartile ranges (IQR) for kit return at T1 and T2 are shown for each cohort.

We then analyzed the temporal variability in the gut microbiome composition over the two time-points of sample collection after SARS-CoV-2 testing in the subset of participants who returned both T1 and T2 samples within the defined timeframes (*n* = 148 paired participants; 296 samples). Sampling timelines and median return times for kits are outlined in Figure 3a. Time to kit receipt did not differ between groups for both timepoints (Tukey HSD, *p* ≥ 0.1; Supplemental Figure 5). Principal coordinate analysis (PCoA) of BC dissimilarities showed significant changes in microbial composition over time within individuals (PERMANOVA, within-individual permutation, *p* = 0.001; Figure 3b) and within each cohort (Figure 3c). However, the extent of these changes does not significantly differ among the three cohorts (Tukey’s HSD *p* > 0.5 for all comparisons; Figure 3d), suggesting that the changes among the two time points represent the normal variability expected over time and are unlikely to underlie the development of LC.

### Gut microbiome composition during acute infection is associated with long COVID

**Figure 4.**
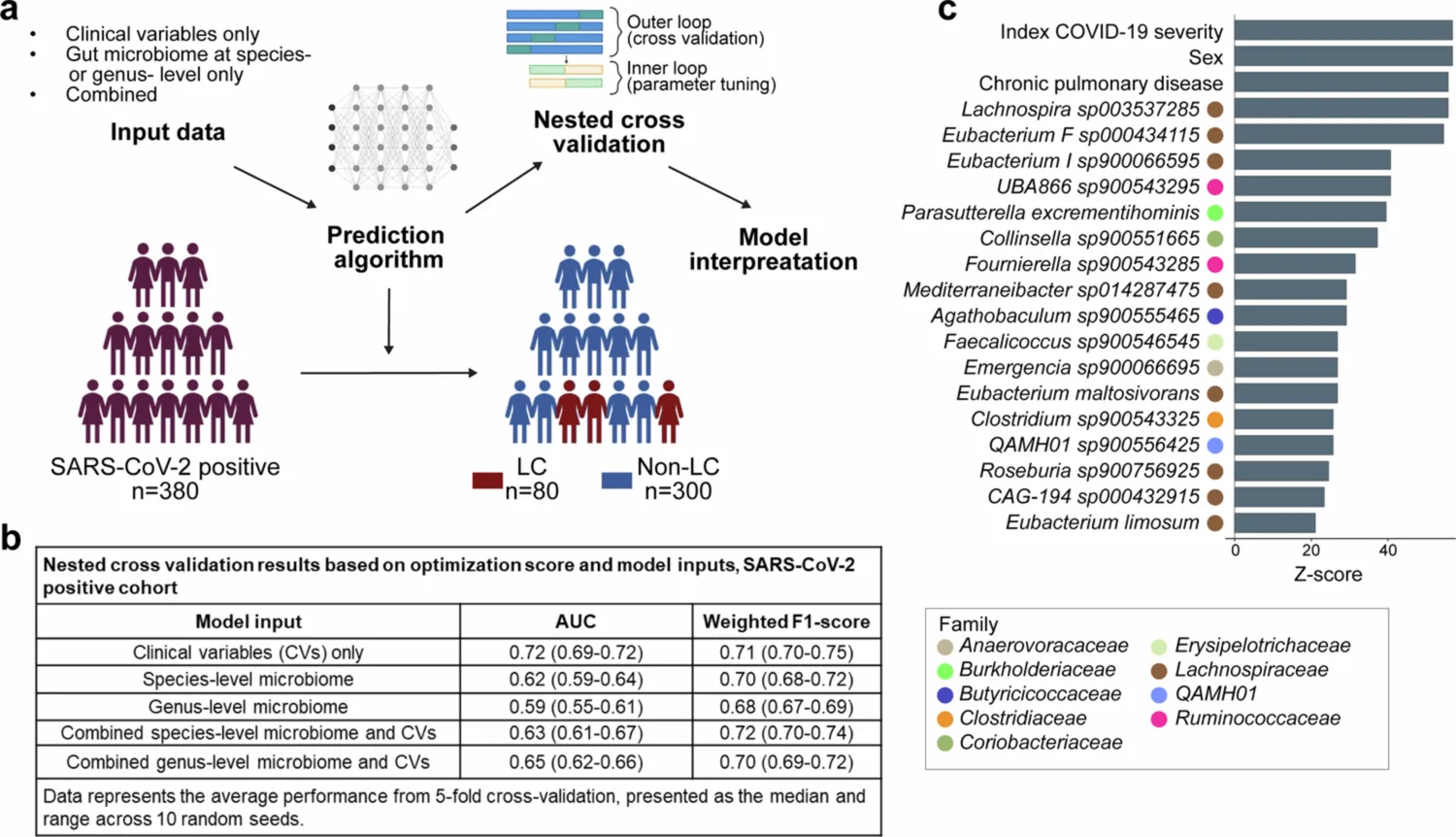
Gut microbiome composition and clinical variables can predict LC in a SARS-CoV-2 positive cohort. (a) Framework for developing and validating predictive algorithm – logistic regression with L1 regularization – for predicting LC within a cohort of COVID-19 patients. Model inputs included clinical variables, and species- and genus-level microbiome abundances, as well as their integration to calibrate each model’s predictive power. (b) Nested cross-validation results per model inputs and model types. (c) Heatmap showing frequencies of the top 20 most impactful features over 50 nested cross-validation iterations, optimized for AUC. This curated list of features consistently influenced model outcomes. Additional information is provided in the Supplemental Methods.

To determine the ability of the gut microbiome to predict LC in 80 individuals from a cohort of 380 SARS-CoV-2-positive patients, we employed logistic regression with L1 regularization (Figure 4a). We initially validated the model using clinical variables or species- and genus-level bacterial abundances independently. Microbiome-only models showed modest discriminatory ability, suggesting that microbiome features contained some predictive signal but were insufficient as classifiers for clinical use. Compared to the clinical variables, microbiome-only models had lower AUCs but comparable weighted F1-scores (Kruskal-Wallis test with Benjamini-Hochberg correction; Figure 4b). The clinical variables only model had a high recall of 0.95 but a lower precision of 0.34, suggesting that clinical variables identified most positive cases but with limited specificity (Supplemental Table 5). We also found that genus-level microbiome models exhibited variable performance depending on the evaluation metric. Combined clinical–microbiome models further supported the contribution of microbiome features alongside clinical factors, although the magnitude of improvement depended on the metric considered. Feature-importance analysis from the combined model identified both clinical characteristics and multiple bacterial taxa among the top-ranked features, including several members of the *Lachnospiraceae* family and other taxonomically diverse organisms (Figure 4c).

### Symptom-based long COVID subphenotypes are associated with distinct gut microbial composition

**Figure 5.**
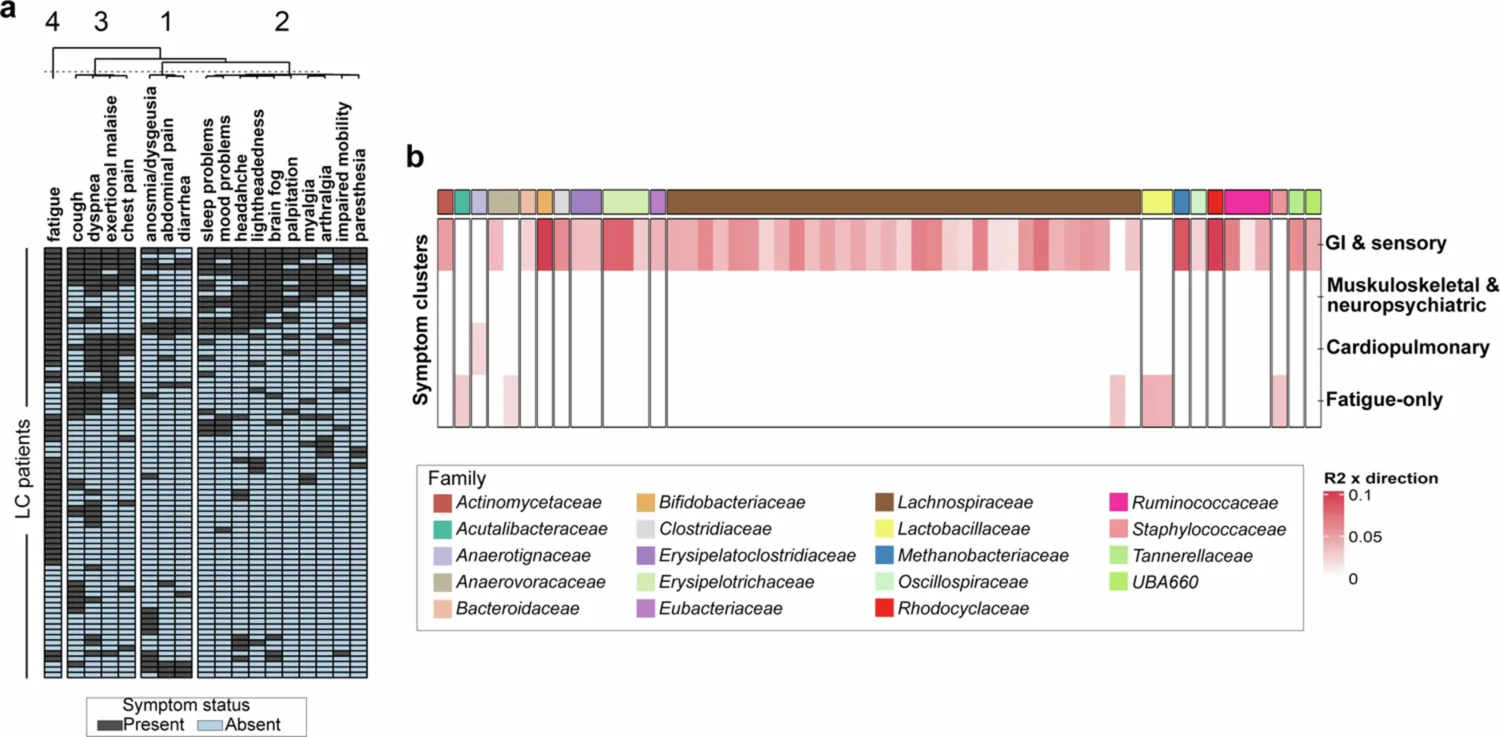
Data-driven clustering of LC symptoms reveals distinct disease subphenotypes associated with unique microbial signatures. (a) Hierarchical and *k*-means clustering of LC symptoms observed in at least 10% of patients revealed four distinct symptom clusters. (b) Differential taxa abundances at the species and genus levels were analyzed between SARS-CoV-2 positive patients with and without individual symptom clusters. Multivariate analysis using ZicoSeq, adjusting for sex in the LC group and for the remaining symptom clusters in each cluster analysis. Taxa with FDR-adjusted *p* < 0.1 are shown.

Long COVID is a heterogeneous entity encompassing symptoms across multiple organ systems. We found fatigue was the most prevalent symptom, reported by 51 individuals, followed by dyspnea (*n* = 36) and cough (*n* = 27). These symptoms were not mutually exclusive. For example, among the 80 patients with LC, 26 (32.5%) experienced only one symptom, while 13 (16%) reported two or three symptoms, and 41 (51%) had more than three symptoms. A subset of patients (16%, *n* = 13) required frequent clinical visits and received specialized care from clinics dedicated to LC management. While the gut microbiome has been associated with the development of LC, it remains unclear if there are specific symptom groups that are more likely to be driven by changes in the gut microbiome. We identified four distinct LC disease subphenotypes based on symptom co-occurrence, each aligning with specific organ systems (Figure 5a): (1) gastrointestinal & sensory (abdominal pain, diarrhea, anosmia/dysgeusia), (2) musculoskeletal & neuropsychiatric (myalgia, arthralgia, impaired mobility, paresthesia, headache, lightheadedness, brain fog, sleep problems, palpitation, and mood issues), (3) cardiopulmonary (dyspnea, chest pain, exertional malaise, and cough), and (4) fatigue-only (fatigue). To determine the potential role of the gut microbiome in LC symptom heterogeneity, we first defined symptom-domain membership for LC patients based on the presence of one or more domain-specific symptoms. Because LC symptoms frequently co-occurred, these domains were intentionally defined as non-mutually exclusive and were used to assess microbiome associations with specific symptom domains rather than to classify patients into discrete patient subtypes. Following this classification, we employed ZicoSeq to analyze species-level gut microbiome composition associated with these symptom domains. For each tested symptom domain, the model was adjusted for sex and for membership in the remaining symptom domains to reduce the possibility that observed microbiome associations were driven by overlapping symptom profiles. We found that the gastrointestinal and sensory cluster exhibited the most prominent changes in taxa abundance, with 50 species, including 30 from the *Lachnospiraceae* family, three each from *Anerovoracaeae*, *Erysipelatoclostridiaceae*, *Erysipelotrichaceae*, and *Ruminococcaceae* families, significantly increased in comparison with SARS-CoV-2 positive patients who do not develop persistent GI/sensory symptoms (FDR adj. *p* < 0.1; Figure 5b). Specifically, the fatigue-only group was associated with multiple genera, including *Lactobacillus*, *Limosilactobacillus*, *Sellimonas*, *Staphylococcus*, *Acutalibacteraceae; UBA1691*, and *Anaerovoracaceae; CAG-145*, whereas there were minimal associations with cardiopulmonary, musculoskeletal, and neuropsychiatric clusters.

## Discussion

In this study, we found that SARS-CoV-2-positive individuals who later developed LC harbored a distinct gut microbiome composition within 1 month of acute infection. To assess its predictive value, we trained an L1-penalized logistic regression. Our analyses revealed that microbiome-only models have modest discriminatory ability, indicating that gut microbiome features capture some signals related to subsequent long COVID risk but are insufficient as stand-alone classifiers for clinical use. Integration of the microbiome composition with clinical variables improves predictive performance for LC. These findings suggest that incorporating the gut microbiome composition and clinical variables may contribute complementary information on host health and immune status at the time of infection.

There is growing recognition that long COVID is a heterogeneous disorder characterized by a wide range of symptoms, likely representing different subphenotypes. A previous study^7^ used topic modeling on newly incident conditions (30–180 d) extracted from EHR and identified four LC subphenotypes, including (1) cardiac and renal, (2) respiratory, sleep, and anxiety, (3) musculoskeletal and nervous, and (4) digestive and respiratory. Another study conducted in China with two cross sectional and one longitudinal cohort studied the role of gut microbiome in long COVID phenotypic heterogeneity and they have found three microbiome-based enterotypes associated with disease heterogeneity.^20^ Our approach differed in that we based our cluster identification on the co-occurrence of symptoms, leading to the identification of distinct system-based separation of long COVID symptoms. We then studied microbiome composition in relation to these clusters and found that they are differentially associated with the gut microbiome. Interestingly, patients developing gastrointestinal/sensory symptoms, and fatigue as part of LC were associated with significant alterations in microbial composition within 1 month of acute infection, while those developing cardiopulmonary and musculoskeletal & neuropsychiatric clusters had minimal associations with specific genera or species. Similar to the recent study,^20^ we found changes in *Lachnospiraceae* were associated with the development of GI symptoms. Interestingly, members of *Lachnospiraceae* family are a one of the major short chain fatty acid producers with proposed impact on the host physiology and gut immunity as well as the disease and health states.^47^ ^,^ ^48^ Long COVID may be conceptually viewed as part of a broader spectrum of chronic post-infectious sequelae, similar to post-infectious functional bowel disorders such as post-infectious irritable bowel syndrome.^49^ ^,^ ^50^ In these conditions, persistent alterations in host-microbiome interactions, among other proposed mechanisms, may contribute to symptoms after the acute infection has resolved.^51^ ^,^ ^52^ However, because our study did not include comparator cohorts with other post-infectious syndromes, we cannot determine whether the microbial patterns observed are specific to LC or shared across chronic post-infectious conditions.

The distinct microbiome profile observed among SARS-CoV-2-positive participants may reflect both pre-existing host-microbiome differences and infection-induced remodeling of the gut ecosystem. Unlike some respiratory viruses that may affect the gut primarily through indirect gut-lung axis mechanisms, SARS-CoV-2 can also directly infect ACE2-expressing intestinal enterocytes^53^ ^,^ ^54^, and prior studies have shown persistence of SARS-CoV-2 RNA and nucleocapsid protein in intestinal mucosal tissue months after acute infection,^12^ supporting the gut as a biologically plausible site of ongoing host-viral-microbiome interaction. However, because non-SARS-CoV-2 respiratory viruses have not been systematically tested in our SARS-CoV-2-negative group, we cannot determine whether these microbiome patterns are unique to SARS-CoV-2 infection or shared with other respiratory viral illnesses.

Several taxa identified in our analyses, including members of *Lachnospiraceae* and *Ruminococcaceae* as well as *Lactobacillus*/*Limosilactobacillus*, have biological relevance through microbial functions involved in carbohydrate fermentation, short-chain fatty acid production, epithelial barrier maintenance, and host immune signaling.^55^ Although our microbiome-based models showed only modest discriminatory performance for LC as a broad heterogeneous outcome, the stronger associations observed in gastrointestinal/sensory and fatigue-only symptom clusters suggest that microbiome changes may contribute selectively to specific LC manifestations rather than serving as a universal LC biomarker. This observation is consistent with prior PACS studies showing persistent gut dysbiosis and phenotype-specific microbiome associations, supporting a potential role for gut-immune, and metabolite-mediated pathways in selected LC presentations.^20^

Although fecal calprotectin levels did not differ between cohorts at T1 or T2, levels decreased by T2 across all cohorts. Given that 91% of SARS-CoV-2-positive participants and 56% of contemporaneous SARS-CoV-2-negative participants were symptomatic during the acute phase, this decrease likely reflects the resolution of acute infectious or inflammatory syndromes rather than an LC-specific signal.^56^

Several strengths of our study highlight its unique contributions. We enrolled a large outpatient cohort with predominantly mild disease, which is more representative of the current post-pandemic population. While prior studies have greatly advanced our understanding, they primarily involved control groups composed of asymptomatic individuals, non-disease controls from colonoscopy trials, or healthy controls recruited pre-pandemic. Our inclusion of a contemporaneous symptomatic control group thus complements the existing knowledge through robust clinical adjustment. Additionally, we manually reviewed all post-SARS-CoV-2 symptoms and conditions, reducing bias toward respiratory manifestations typical of methods relying on ICD codes.^2^ Finally, we developed and validated machine-learning models that sequentially integrated clinical variables and gut-microbiome profiles, quantifying the microbiome’s incremental predictive value, and improving model interpretability and robustness.

Despite these strengths, our study has several limitations. While this represents one of the largest cohort studies involving outpatient COVID-19 patients, the subset of LC patients (*n* = 80) was relatively modest. Nevertheless, the ML models we employed showed promise by capturing signals that could associate with LC using only microbiome data, as indicated by cross-validation metrics. However, to increase confidence in these findings, they require external validation. The lack of a comparable outpatient dataset with microbiome sampling during acute infection restricts our ability to validate our results using previously published studies (Supplemental Table 6). Although our study captured several important potential clinical confounders, dietary information was not available. Because a habitual and recent diet can influence the gut microbiome composition,^57^ residual confounding by diet cannot be excluded. Accordingly, the LC subphenotype analyses were exploratory and may be subject to residual confounding by age, comorbidities, antibiotic exposure, and other clinical factors. In addition, because the paired T1‒T2 analysis included a smaller subset of participants and a relatively short follow-up interval, these findings should be interpreted as reflecting early temporal microbiome changes. Future studies with larger cohorts and longitudinal sampling over longer periods will help build on our findings.

## Conclusion

Our results demonstrate that the gut microbiome during acute SARS-CoV-2 infection can potentially identify patients at risk of developing LC. Variations in the gut microbiome may be intricately linked to the pathophysiology of specific LC symptoms, including gastrointestinal/sensory and fatigue-only clusters. Future studies incorporating the microbiome are needed to refine ML-based predictive models, further validate findings from our study, and assess potential microbiome-driven mechanisms that underlie the development of symptoms in LC patients.

## Supplementary Material

Supplementary Material Supplemental Materials.docx

## Data Availability

Source Data are provided with this paper. The microbiome data are publicly available on NCBI's BioProject database with BioProject ID PRJNA1167328. The SARS-CoV-2 genome sequences from this study have been deposited in the NCBI GenBank database under accession codes PV201775–PV201814 and SAMN47241892–SAMN47241893.
