# Supplementary material for: Gut microbiome signatures during acute infection are associated with long COVID
Source: Gut Microbes. 2026 Aug 19;18(1):2718581. doi: 10.1080/19490976.2026.2718581 (PMC13502034; doi:10.1080/19490976.2026.2718581)
Supplement: Supplementary file 1 [file KGMI_A_2718581_SM0769.docx]

Supplemental Materials for “**Gut Microbiome Signatures During Acute Infection Predict Long COVID”**

Isin Y. Comba^1^, Ruben A. T. Mars^2,3^, Lu Yang^3,4^, Mitchell Dumais^5^, Jun Chen^3, 4^, Trena M. Van Gorp^3^, Jonathan J. Harrington^3^, Jason P. Sinnwell^4^, Stephen Johnson^4^, LaRinda A. Holland^6^, Adam K. Khan^6^, Efrem S. Lim^6^, Christopher Aakre^5^, Arjun P. Athreya^7^, Georg K. Gerber^8,9,10^, Jack C. O’Horo^1,11^, Konstantinos N. Lazaridis^2^, Purna C. Kashyap^2,12,13*^

1 Division of Public Health, Infectious Diseases, and Occupational Medicine, Mayo Clinic, Rochester, MN, 55905, US

2 Division of Gastroenterology and Hepatology, Mayo Clinic, Rochester, MN, 55905, USA

3 Center for Individualized Medicine, Mayo Clinic, Rochester, MN, 55905, USA

4 Department of Quantitative Health Sciences, Mayo Clinic, Rochester, MN, 55905, USA.

5 Department of Medicine, Mayo Clinic, Rochester, MN, 55905, US

6 Center for Fundamental and Applied Microbiomics, the Biodesign Institute, Tempe, Arizona, USA.

7 Department of Molecular Pharmacology and Experimental Therapeutics, Mayo Clinic, Rochester, Minnesota, USA

8 Division of Computational Pathology, Brigham and Women's Hospital, Boston, MA, 02115, USA

9 Health Sciences and Technology, Harvard University and MIT, Cambridge, MA, 02139, USA

10 Harvard Medical School, Boston, MA, USA

11 Division of Pulmonary and Critical Care Medicine, Mayo Clinic, Rochester, MN, USA

12 Department of Physiology and Biomedical Engineering, Mayo Clinic, Rochester, MN, USA

13 Lead contact

***Correspondence:** [kashyap.purna@mayo.edu](mailto:kashyap.purna@mayo.edu)

**Supplemental Figures:**

**Supplemental Figure 1. Determining the optimal number of clusters for analyzing disease heterogeneity. a** Multiple correspondence analysis (MCA) plot of variables (symptoms) illustrating the relationships between symptoms categorized as "Present" (purple) and "Absent" (yellow) in the binary dataset. Symptoms clustering in similar regions indicate shared patterns or associations. The length and direction of each arrow indicate the strength and orientation of the symptom’s contribution to the variability captured in first two dimensions. **b** The within-cluster sum of squares (WCSS) for *k*-means clustering of LC symptoms was plotted against numbers of clusters (upper panel), along with the corresponding percentage decrease in WCSS (lower panel). An "elbow" in the plot was seen at *k*=4, suggesting that four clusters optimally balance cluster cohesion and parsimony. **c** Mean silhouette widths s(*i*) were calculated for *k* values ranging from 2 to 10. The silhouette width for each symptom measures how well it fits within its own cluster compared to others; with higher values closer to one indicating the no. of cluster providing the more cohesive grouping of the data. The average silhouette width peaks at *k*=4.

**Supplemental Figure 2. T1 kit return timeline.** Cumulative proportion of T1 stool samples returned in days since the index SARS‑CoV‑2 test, stratified by non‑LC, LC, SARS‑CoV‑2–negative participants, and all T1 samples. Dashed and dotted vertical lines denote 15 and 30 days post–index test, respectively.

**Supplemental Figure 3. Additional analysis for Figure 2. a** Alpha diversity comparisons among LC, non-LC, and SARS-CoV-2-negative groups using the Shannon index. Statistical significance was assessed by one-way ANOVA. Boxplots display the median (central line), interquartile range (25th and 75th percentiles; box), and whiskers extending to the 5th and 95th percentiles. **b** Beta diversity measured by generalized UniFrac distances at the species level. The statistics inlet were generated using PERMANOVA, n=999. Analysis is adjusted for age, sex, baseline Charlson’s Comorbidity Index (CCI) score, chronic pulmonary disease and 30-day antibiotic use.

**Supplemental Figure 4. Comparative analysis of microbial functional pathway abundances. a** Differential abundance analysis of microbial pathways using ZicoSeq highlighting nominal associations between LC (n=80) and non-LC (n=299) cohorts compared to SARS-CoV-2-negative (n=419) adjusted for clinical confounders. Level of significance are represented by Roman numerals: I, FDR-adjusted *p*<0.1; II, *p*<0.05; and III, for *p*<0.01. **b** Comparison of individual microbial pathways. In all panels, boxplots represent data from non-LC and SARS-CoV-2-negative cohorts. The central line indicates the median, and the box represents the interquartile range (IQR). Whiskers extend to data points within 5^th^ and 95^th^ percentile. FDR-adjusted *p*-values were generated using ZicoSeq.

**Supplemental Figure 5. Timing of stool kit returns in the longitudinal paired cohort (n=148).** Median values and interquartile ranges are shown for each time point.

**Supplemental Tables:**

| **Supplemental Table 1. Clinical characteristics of the study population** | | | | |
| --- | --- | --- | --- | --- |
|  | LC (n=80) | Non-LC (n=300) | SARS-CoV-2-negative (n=419) | *p* |
| Chronic pulmonary disease | 21 (26) | 32 (11) | 68 (16) | 0.003 |
| Diabetes mellitus | 9 (11) | 22 (7) | 37 (9) | 0.5 |
| Chronic kidney disease | 3 (4) | 24 (8) | 30 (7) | 0.4 |
| Moderate to severe liver disease | 0 | 4 (1) | 0 | - |
| Myocardial infarction | 2 (2.5) | 3 (1) | 9 (2) | 0.4 |
| Rheumatological disease | 4 (5) | 8 (3) | 20 (5) | 0.3 |
| Metastatic cancer | 3 (4) | 7 (2) | 13 (3) | 0.6 |
| Inflammatory bowel disease | 4 (5) | 8 (3) | 10 (2) | 0.4 |
| Transplant history | 2 (2.5) | 6 (2) | 4 (1) | 0.3 |
| Solid organ transplant | 2 (2) | 3 (1) | 3 (1) |  |
| Bone marrow transplant | 0 | 3 (1) | 1 (0.2) |  |
| Proton pump inhibitor use | 20 (25) | 58 (19) | 95 (23) | 0.4 |
| Immunosuppressive medication | 7 (9) | 13 (4) | 30 (7) | 0.2 |
| Glucocorticoids | 1 (1) | 3 (1) | 8 (2) |  |
| Antimetabolites | 1 (1) | 1 (0.3) | 8 (2) |  |
| Cancer chemotherapeutics | 1 (1) | 1 (0.3) | 6 (1) |  |
| Calcineurin inhibitors | 2 (2.5) | 2 (1) | 1 (0.2) |  |
| Antiproliferative agents | 1 (1) | 5 (2) | 4 (1) |  |
| Biologics | 3 (4) | 6 (2) | 6 (1) |  |
| Other | 1 (1) | 1 (0.3) | 2 (0.5) |  |
| Data is represented as n (%). *P*-values were generated using Pearson's Chi-squared test, or Fisher's exact test. Comorbidities and medication use history of three SARS-CoV-2-negative controls are missing (same patients across variables). Immunosuppressive medication use within 30 days prior to SARS-CoV-2 test date. Glucocorticoid use was defined as the equivalent to >7.5 mg total daily prednisone for a minimum of four weeks. | | | | |

| **Supplemental Table 2. Distribution of symptom types, index SARS-CoV-2 testing in the symptomatic cohort** | | | |
| --- | --- | --- | --- |
| Symptom type, index testing, n (%) | LC (n=79) | Non-LC (n=268) | SARS-CoV-2-negative (n=234) |
| Cough | 57 (72) | 168 (63) | 73 (31) |
| Headache | 46 (58) | 146 (54) | 73 (31) |
| Myalgia | 36 (46) | 117 (44) | 44 (19) |
| Rhinorrhea/nasal congestion | 38 (48) | 107 (40) | 20 (8.5) |
| Fever | 26 (33) | 92 (34) | 41 (18) |
| Fatigue | 35 (44) | 72 (27) | 42 (18) |
| Chills | 24 (30) | 81 (30) | 36 (15) |
| Sore throat | 23 (29) | 77 (29) | 75 (32) |
| Change or loss of taste | 26 (33) | 61 (23) | 6 (3) |
| Change or loss of smell | 21 (27) | 61 (23) | 3 (1) |
| Dyspnea | 26 (33) | 46 (17) | 32 (14) |
| Chest distress | 29 (37) | 36 (13) | 16 (7) |
| Diarrhea | 20 (25) | 44 (16) | 38 (16) |
| Nausea | 17 (22) | 24 (9) | 45 (19) |
| Sputum production | 15 (19) | 25 (9) | 14 (6) |
| Lightheadedness | 16 (20) | 22 (8) | 7 (3) |
| Abdominal pain | 8 (10) | 12 (4) | 11 (5) |
| Vomiting | 5 (6) | 7 (3) | 19 (8) |
| Other | 20 (25) | 21 (8) | 34 (15) |
| Missing | 11 (14) | 29 (11) | 67 (29) |

| **Supplemental Table 3. Provider-assigned or suspected etiologies and documentation status among symptomatic SARS-CoV-2-negative participants** | |
| --- | --- |
| Documented etiology | n=234 |
| Sinusitis, including viral, bacterial, or non-infectious sinusitis | 17 |
| Viral upper respiratory infection | 13 |
| Newly diagnosed other chronic conditions, including carcinoid tumor, irritable bowel syndrome, small bowel obstruction, palpitations, or other diagnoses | 9 |
| Gastroenteritis (viral, parasitic, or foodborne) | 7 |
| Community-acquired pneumonia (viral, bacterial, or fungal) | 7 |
| Vertigo (Bening positional paroxysmal vertigo) | 6 |
| COPD or asthma exacerbation | 5 |
| Pharyngitis (viral, *group B* *streptococcal*, or abscess) | 5 |
| Atypical chest pain | 5 |
| Headache disorders (migraine etc,) | 4 |
| Urinary tract infection (uncomplicated, or pyelonephritis) | 4 |
| Ear infection (acute otitis externa, otitis media, or mastoiditis) | 3 |
| Other infections (anaplasmosis, infectious mononucleosis, cellulitis) | 3 |
| Drug side effect | 2 |
| Unknown; no provider assessment directed at reported symptoms | 121 |
| Not available; no longitudinal follow-up | 13 |
| Screening indication with reported symptoms | 10 |

| **Supplemental Table 4. Additional stool characteristics and SARS-CoV-2 genotype of the study population, all cohorts** | | | | | | |
| --- | --- | --- | --- | --- | --- | --- |
|  | Timepoint-1 | | | Timepoint-2 | | |
|  | LC (n=80) | Non-LC (n=300) | SARS-CoV-2-negative (n=419) | LC (n=27) | Non-LC (n=73) | SARS-CoV-2-negative (n=48) |
| Bristol score |  |  |  |  |  |  |
| 1 | 4 (5) | 5 (2) | 12 (3) | 2 (7) | 2 (3) | 1 (2) |
| 2 | 7 (9) | 15 (5) | 29 (7) | 1 (4) | 6 (8) | 3 (6) |
| 3 | 9 (11) | 38 (13) | 64 (15) | 5 (18.5) | 5 (7) | 8 (17) |
| 4 | 23 (29) | 94 (31) | 124 (30) | 7 (26) | 23 (31.5) | 12 (25) |
| 5 | 4 (5) | 16 (5) | 23 (5.5) | 2 (7) | 5 (7) | 2 (4) |
| 6 | 7 (9) | 31 (10) | 38 (9) | 0 | 8 (11) | 5 (10) |
| 7 | 3 (4) | 6 (2) | 2 (0.5) | 0 | 0 | 0 |
| Missing | 23 (29) | 95 (32) | 127 (30) | 10 (37) | 24 (33) | 17 (35) |
| SARS-CoV-2 genotype |  |  |  |  |  |  |
| AY.100 | - | 2 (1) | - | - | - | - |
| AY.103 | - | 3 (1) | - | - | - | - |
| AY.25 | - | 1 (0.3) | - | - | - | - |
| AY.3 | - | 2 (1) | - | - | - | - |
| AY.44 | - | 1 (0.3) | - | - | - | - |
| AY.47 | 1 (1) | - | - | - | - | - |
| AY.54 | - | 1 (0.3) | - | - | - | - |
| B.1 | - | 1 (0.3) | - | - | - | - |
| B.1.1.222 | - | 1 (0.3) | - | - | - | - |
| B.1.2 | 2 (2.5) | 9 (3) | - | - | - | - |
| B.1.234 | 1 (1) | 1 (0.3) | - | - | - | - |
| B.1.239 | - | 1 (0.3) | - | - | - | - |
| B.1.243 | - | 1 (0.3) | - | - | - | - |
| B.1.311 | 1 (1) | - | - | - | - | - |
| B.1.427 | - | 1 (0.3) | - | - | - | - |
| B.1.429 | - | 1 (0.3) | 1 (0.2) | - | - | - |
| B.1.565 | - | 1 (0.3) | - | - | - | - |
| B.1.617.2 | - | 5 (2) | - | - | - | - |
| Unassigned (e.g., poor coverage, high ambiguity, conflicting lineage) | 1 (1) | 3 (1) | 2 (0.4) | - | 1 (1) | - |
| Categorical variables are reported as n (%). Genomic lineages of SARS-CoV-2 sequences were determined using the Phylogenetic Assignment of Named Global Outbreak Lineage (Pangolin) | | | | | | |

| **Supplemental Table 5. Nested cross validation results based on optimization score and model inputs, SARS-CoV-2 positive cohort** | | |
| --- | --- | --- |
| **Model input** | **Precision** | **Recall** |
| Clinical variables (CVs) only | 0.34 (0.31-0.37) | 0.95 (0.90-0.96) |
| Species-level microbiome | 0.30 (0.23-0.32) | 0.44 (0.40-0.48) |
| Genus-level microbiome | 0.26 (0.20-0.29) | 0.42 (0.36-0.45) |
| Combined species-level microbiome and CVs | 0.35 (0.27-0.48) | 0.46 (0.41-0.49) |
| Combined genus-level microbiome and CVs | 0.32 (0.28-0.38) | 0.49 (0.43-0.51) |
| Data represents the average performance from 5-fold cross-validation, presented as the median and range across 10 random seeds. | | |

| **Supplemental Table 6 – Overview of studies in gut microbiome and long COVID** | | | | |
| --- | --- | --- | --- | --- |
| **Year, author** | **SARS-CoV-2 positive cohort size (no. of patients)** | **Hospitalization status** | **Sequencing method** | **Timing of stool collection** |
| Liu Q et al (2022) [1] | 133 | Inpatient during acute infection | Shotgun metagenomic sequencing | At hospital admission;  3 months and 6 months after respiratory SARS-CoV-2 viral clearance. |
| Liu Q, Mak JWY, Su Q, et al. (2022) [2] | 106 | Inpatient during acute infection | Shotgun metagenomic sequencing | At hospital admission; 1 month and 6 months after the hospital discharge |
| Zhang et al. (2023) [3] | 187 | Inpatient during acute infection | 16S rRNA gene sequencing (V3–V4) | 12 (+/- 1) months after the hospital discharge |
| Su Q et al. (2024) [4] | 196 | Inpatient during acute infection | Shotgun metagenomic sequencing | During hospitalization |

**Supplemental Methods**

**Study population**

Negative controls underwent testing based on a provider-initiated screening questionnaire due to exposure or new-onset symptoms, or as part of routine preprocedural, travel, or other screenings. The questionnaire included exposure to a suspected or confirmed COVID-19 patient within 72 hours or new-onset symptoms (**supplementary table 2**) not explained by an existing condition. Of the initial 1,061 participants recruited, 242 were excluded due to failure to return kits, withdrawal from the study, delayed kit returns, failed sequencing, or incomplete medical records. The remaining 819 participants were followed prospectively with review of EHR for at least 12 months. An additional 20 participants, where adequate clinical follow-up was not available were excluded. One individual who initially tested negative but received a positive result two days later was reclassified into the SARS-CoV-2–positive cohort. Additionally, two participants from the SARS-CoV-2–negative cohort tested positive approximately one year after enrollment and subsequently developed long COVID. Due to the small number of such cases (n=2), they were not analyzed as a separate cohort. The final analysis included 947 stool samples from 799 subjects (380 SARS-CoV-2 positive and 419 SARS-CoV-2 negative).

**Microbial DNA extraction**

Briefly, PowerBead Pro plates were loaded in a TissueLyser II for homogenization before loading samples onto a QIAcube HT for automated DNA extraction. This protocol was selected because it includes both chemical and mechanical lysis, which have been shown to be important for accurate recovery of abundances of difficult-to-lyse bacteria (e.g., some Gram-positive species), and allows for automation, which has been shown to reduce batch variability effects. DNA was quantified using Qubit.

**Statistical analysis**

*Descriptive statistics*

Descriptive statistics were reported using frequencies and proportions for categorical data and mean, median, standard deviation (SD), and interquartile range (IQR) for continuous variables. Categorical data were analyzed using the chi-square (χ2) test or Fisher's exact test, while group comparisons for continuous variables were conducted using Student’s *t*-test or Wilcoxon–Mann–Whitney test. Assessment of clinical data completeness revealed that missing data were minimal, constituting less than 0.07% of the entire dataset per respective variables used to adjust microbiome analysis and as model input. Missing values were imputed using mean, median, or mode methods, depending on the data type. Statistical significance was set at *p*<0.05, and all statistical analyses were performed using R.

*Microbiome data analysis*

For microbiome data pre-processing, 10% prevalence, 0.01% mean and 0.2% maximum abundance filters were applied. Outliers were handled using winsorization, capping the top 3% of data points, thereby minimizing the influence of extreme values. Zeroes were imputed through posterior sampling, which was conducted 25 times to reduce the variability of results. Data were transformed using square root transformation. The statistical significance was assessed using permutation-based false discovery rate (FDR). The analysis also included reference-based multiple-stage normalization to address compositional effects, using six normalization stages with a reference set comprising 50% of the samples. Differential abundance analysis was conducted using ZicoSeq package in R [5]. Metagenomics functional pathways were analyzed using 10% prevalence and 0.2% maximum abundance filtering followed by the same statistical tools at the same set up. Pathway types were determined using ontology-based MetaCyc pathway classification [6]. Data visualization for microbial taxa and functional pathway comparisons were adopted from standard biostatistical tool [7–9].

**Software and algorithms**

Input data types included species-level (n=1318) and genus-level (n=650) relative abundance matrices, and clinical variables.

*Data pre-processing*

No feature selection was applied to clinical variables. Microbiome data was filtered by removing species-level taxa with <100 reads and genus-level taxa with <10 reads in more than 10% of subjects (n=38). Relative abundances were calculated from filtered microbiome data and subsequently transformed using center-log transformation to account for compositionality. Categorical clinical variables were transformed into binary matrices via one-hot encoding. Finally, numerical variables from both clinical and microbiome datasets were normalized to a 0-1 scale using MinMaxScaler().

*Model development and nested cross-validation*

*Logistic regression with L1 regularization:* Logistic regression from scikit-learn (v1.2.2) was employed with the ‘liblinear’ solver [10]. The model was configured to incorporate the computed class weights to correct for unbalanced classes. Hyperparameter tuning was performed via GridSearchCV during nested cross-validation, optimizing the regularization strength parameter *C* over 100 evenly spaced values on a logarithmic space, covering multiple orders of magnitude from 10^-3^ to 10^1^ (0.001 to 10). The scoring metrics for model optimization were AUROC (Area Under the Receiver Operating Characteristic), weighted F1 score, precision and recall.

*Model interpretation*

To determine the impact of individual bacterial taxa on model output from logistic regression, coefficients were derived from log-odds. For these experiments, we chose the models integrating species-level microbiome data and clinical variables. To assess consistency and repeatability of the top 20 most influential predictors, the analysis was structured around 10 random seeds coupled with 5-fold cross-validation in the outer loop, which resulted in 50 runs per model. Statistically, each predictor was treated as having an equal probability of being selected as one of the top 20 most influential features The discrete random variable X=1 indicated a feature’s selection as one of the top 20 most influential predictors vs. X=0 otherwise. Given the large number of predictors (n=1,318) and low probability of any single predictor being selected, Poisson distribution was employed to approximate the binomial distribution. Z-scores were calculated for each predictor to assess deviation from the expected frequency, aiding in their ranking based on occurrence.

*n=*50 (Bernoulli trials)

*x=*no. of appearance in top 20 for each predictor

*p=*probability of the event (20/1318)

Probability mass function of Poisson distribution

P(X=x) = $\frac{\lambda^{x}ⅇ^{-\lambda}}{x!}$

(1)

(2)

λ = np = μ = σ^2^

(3)

z score = (x- λ)/sqrt(x)

**References**

1.	Liu Q, Su Q, Zhang F, Tun HM, Mak JWY, Lui GC-Y, et al. Multi-kingdom gut microbiota analyses define COVID-19 severity and post-acute COVID-19 syndrome. Nat Commun. 2022;13: 6806. doi:10.1038/s41467-022-34535-8

2.	Liu Q, Mak JWY, Su Q, Yeoh YK, Lui GC-Y, Ng SSS, et al. Gut microbiota dynamics in a prospective cohort of patients with post-acute COVID-19 syndrome. Gut. 2022;71: 544–552. doi:10.1136/gutjnl-2021-325989

3.	Zhang D, Zhou Y, Ma Y, Chen P, Tang J, Yang B, et al. Gut Microbiota Dysbiosis Correlates With Long COVID-19 at One-Year After Discharge. J Korean Med Sci. 2023;38: e120. doi:10.3346/jkms.2023.38.e120

4.	Su Q, Lau RI, Liu Q, Li MKT, Yan Mak JW, Lu W, et al. The gut microbiome associates with phenotypic manifestations of post-acute COVID-19 syndrome. Cell Host Microbe. 2024;32: 651-660.e4. doi:10.1016/j.chom.2024.04.005

5.	Yang L, Chen J. A comprehensive evaluation of microbial differential abundance analysis methods: current status and potential solutions. Microbiome. 2022;10: 130. doi:10.1186/s40168-022-01320-0

6.	Caspi R, Billington R, Ferrer L, Foerster H, Fulcher CA, Keseler IM, et al. The MetaCyc database of metabolic pathways and enzymes and the BioCyc collection of pathway/genome databases. Nucleic Acids Res. 2016;44: D471–D480. doi:10.1093/nar/gkv1164

7.	Shetty A. Sudarshan, Lahti Leo. microbiomeutilities: Utilities for Microbiome Analytics. 2020. Available: http://microbiome.github.com/microbiome

8.	Barnett D, Arts I, Penders J. microViz: an R package for microbiome data visualization and statistics. J Open Source Softw. 2021;6: 3201. doi:10.21105/joss.03201

9.	Hadley Wichkham. ggplot2: Elegant Graphics for Data Analysis. New York: Springer-Verlag; 2016.

10.	Pedregosa F, Varoquaux G, Gramfort A, Michel V, Thirion B, Grisel O, et al. Scikit-learn: Machine Learning in Python. arXiv; 2018. doi:10.48550/arXiv.1201.0490

11.	Abadi M, Agarwal A, Barham P, Brevdo E, Chen Z, Citro C, et al. TensorFlow: Large-Scale Machine Learning on Heterogeneous Distributed Systems. arXiv; 2016. doi:10.48550/arXiv.1603.04467
